# Effects of Essential Fatty Acid Supplementation on *in vitro* Fermentation Indices, Greenhouse Gas, Microbes, and Fatty Acid Profiles in the Rumen

**DOI:** 10.3389/fmicb.2021.637220

**Published:** 2021-03-11

**Authors:** Sardar Muhammad Amanullah, Dong Hyeon Kim, Dimas Hand Vidya Paradhipta, Hyuk Jun Lee, Young Hoo Joo, Seong Shin Lee, Eun Tae Kim, Sam Churl Kim

**Affiliations:** ^1^Division of Applied Life Science (BK21Four), Institute of Agriculture and Life Science, Gyeongsang National University, Jinju, South Korea; ^2^Biotechnology Division, Bangladesh Livestock Research Institute, Savar, Bangladesh; ^3^Dairy Science Division, National Institute of Animal Science, Cheonan, South Korea; ^4^Faculty of Animal Science, Universitas Gadjah Mada, Yogyakarta, Indonesia

**Keywords:** biohydrogenation, fatty acid, methane emission, rumen fermentation, rumen microbes

## Abstract

This study estimated the effect of essential fatty acid (FA) supplementation on fermentation indices, greenhouse gases, microbes, and FA profiles in the rumen. The treatments used pure FAs consisting of C18:2n-6 FA (LA), C18:3n-3 FA (LNA), or a mixture of these FAs at 1:1 ratio (Combo). *In vitro* rumen incubation was performed in 50 mL glass serum bottles containing 2 mg of pure FAs, 15 mL of rumen buffer (rumen fluid+anaerobe culture medium = 1:2), and 150 mg of synthetic diet (411 g cellulose, 411 g starch, and 178 g casein/kg dry matter) at 39°C for 8 h with five replications and three blanks. In rumen fermentation indices, LA exhibited highest (*P* < 0.05) ammonia-N and total gas volume after 8 h of incubation. Furthermore, LA presented lower (*P* < 0.05) pH with higher (*P* < 0.05) total volatile fatty acid (*P* = 0.034) than Combo, while LNA was not different compared with those in the other treatments. Additionally, Combo produced highest (*P* < 0.05) CO_2_ with lowest (*P* < 0.05) CH_4_. In the early hours of incubation, LA improved (*P* < 0.005) *Fibrobacter succinogenes* and *Ruminococcus flavefaciens*, while LNA improved (*P* < 0.005) *Ruminococcus albus*. After 8 h of incubation, LNA had lower (*P* < 0.05) methanogenic archaea than LA and Combo but had higher (*P* < 0.05) rumen ciliates than LA. *R. albus* was higher (*P* < 0.05) in LA than in LNA and Combo. It was observed that the rate of biohydrogenation of n-6 and n-3 FAs was comparatively lowest (*P* < 0.05) in Combo, characterized by higher C18:2n-6 and/or C18:3n-3 FA and polyunsaturated FA (PUFA) concentrations with lower (*P* < 0.05) concentrations of C18:0 and saturated FA and the ratio of saturated FAs to PUFAs. Therefore, this study concluded that dietary C18:2n-6 could improve populations of fibrolytic bacteria and rumen fermentation indices, but dietary mixture of pure C18:2n-6 and C18:3n-3 is recommended because it is effective in reducing enteric methane emissions and resisting biohydrogenation in the rumen with less effect on rumen microbes.

## Introduction

Oil-rich supplements have purposes to manipulate rumen ecosystem through a number of mechanisms, including reductions in organic matter fermentation, rumen ciliate numbers, methanogenic activity, and the use of hydrogen for biohydrogenation (Beauchemin et al., 2009). In addition, the rumen fermentation pattern is significantly influenced by the type of fat (Machmüller et al., 1998). Essential fatty acids (FAs) consisting of linolenic acid (C18:3n-3) and linoleic acid (C18:2n-6) are polyunsaturated FAs (PUFAs) that are required for animals because they are not synthesized in the body. In human, the essential FAs present beneficial effects for human health, such as decreasing cardiovascular morbidity, hypertension, and diabetes mellitus. The C18:3n-3 and C18:2n-6 in linseed and sunflower oils, respectively, have been reported to depress rumen methanogenesis (Machmüller et al., 1998, 2000; Martin et al., 2006; Beauchemin et al., 2009). Methane (CH_4_) emissions by ruminants produce around 5.55% of total global greenhouse gas emission from human activity (FAO, 2016). Additionally, CH_4_ represents a significant energy loss of ruminants in the range of 2–12% of gross energy intake (Johnson and Johnson, 1995; FAO, 2016).

In ruminants, dietary essential FAs undergo biohydrogenation to saturated fatty acids (SFAs) in the rumen, which can be an alternative approach to reduce the amount of free hydrogen for methanogenesis (Machmüller et al., 1998, 2000; Martin et al., 2006; Beauchemin et al., 2009). Additionally, dietary essential FAs have been repeatedly demonstrated to have adverse effects on rumen microbes such as protozoa and methanogenic archaea (Doreau and Ferlay, 1995) and could depress methanogenesis (Fievez et al., 2003). As a negative effect of dietary unsaturated fatty acids (UFAs), Zhang et al. (2008) reported that oil-rich supplement containing C18:2n-6 and C18:3n-3 could inhibit the growth of fibrolytic fungi, *Fibrobacter succinogenes*, and *Ruminococcus flavefaciens* in the rumen but suppressed the methanogenesis. On the other study, dietary soybean oil and linseed oil potentially decrease the populations of *Butyrivibrio fibrisolvens*, *Ruminococcus albus*, and *F. succinogenes*, and also decrease total volatile fatty acid (VFA) production in the rumen (Yang et al., 2009). As a positive effect, Jouany et al. (2008) indicated that C18:3n-3 FA had a greater effect on reducing CH_4_ production than did C18:2n-6 by *in vitro* measurement. Otherwise, dietary UFAs usually undergo extensive biohydrogenation by rumen microbes (Beam et al., 2000), which is the major challenge for achieving the targeted effects of these FAs through dietary supplementation. The extent of biohydrogenation of long-chain PUFAs varies largely depending on the source. A disappearance of C18:2n-6 of approximately 90–98% occurred after 9 h of incubation when it was supplied in a pure form (Honkanen et al., 2012). On the other hand, another study (Carriquiry et al., 2008) observed that approximately 40–65% of the C18:2n-6 fraction could remain after 36 h of incubation when commercial mixed fat sources were used. These results mean that the extent of biohydrogenation of PUFAs in the rumen may vary depending on the form of fat, which may further affect the fermentation pattern. Most of the studies regarding FA supplementation in ruminants were conducted by oil or oil seed sources of FAs (Machmüller et al., 1998, 2000; Martin et al., 2006; Beauchemin et al., 2009). However, the effect of single C18 n-6 or n-3 FA supplementation on rumen ecosystem has been scarcely studied, which might show different effects from C18 n-6 FA and C18 n-3 FA on rumen fermentation, methanogenesis, and microbial population. Especially, the effects of single C18 n-6 or n-3 FA supplementation on population of fibrolytic bacteria were in limited information. Therefore, this study was conducted to estimate the effect of pure C18:2n-6, C18:3n-3, or their mixture on rumen microbes with its fermentation indices, greenhouse gases, and FA profiles.

## Materials and Methods

### *In vitro* Incubation

Animal care and handling in the present study was approved by the Animal Ethics Committee of Gyeongsang National University, South Korea (GNU-191011-E0050). Rumen fluid was collected from two rumen-cannulated Hanwoo heifers (Average body weight = 432.63 kg) fed rice straw (CP = 5.40%, NDF = 63.85%) and concentrate (CP = 12.51%, NDF = 47.51%) mixture at a 8:2 ratio of dry matter (DM) weight. The concentrate mainly consisted of corn meal, soybean meal, soybean hull, and cotton seed pellet. This fluid was collected before morning feeding, filtered with double cheese cloths, stirred to obtain a uniform fluid, and then mixed with anaerobic culture medium at a 1:2 ratio to make rumen buffer. The detail protocol to prepare a rumen buffer was described by Adesogan et al. (2005). Carbon dioxide (CO_2_) gas was flushed into rumen buffer continuously to maintain anaerobic conditions (Carriquiry et al., 2008; Jayanegara et al., 2015). A fat-free synthetic diet was prepared using as a substrate, containing 411 g cellulose (Sigma, C6413), 411 g starch (Sigma, S4180), and 178 g casein (Sigma, C3400) per kg of DM (Carriquiry et al., 2008). The treatments consisted of pure C18:2n-6 FA (Sigma, L1376; LA), pure C18:3n-3 FA (Sigma, L2376; LNA), or a mixture of these FAs (Combo) at 1:1 ratio (*w*/*w*). Five hundred milligrams of LA and LNA was dissolved with 5 mL methanol (A 4524, Fisher Chemical), respectively. After then, 2 mL from each dissolved FAs were mixed for Combo. Before ruminal incubation, 20 μL of dissolved FA (2 mg of FA) was applied into 150 mg of synthetic diet. This synthetic diet applied FA was stored at room temperature to evaporate methanol for 12 h. In the present study, the application rate of each FA treatment was 1.3% DM, which demonstrate the general C18:2n-6 FA and C18:3n-3 FA concentrations in ruminant diet (Kim et al., 2007, 2016). Ruminal incubation was performed in the 50-mL glass serum bottle containing 15 mL of rumen buffer with synthetic diet applied FA at 39°C for 0, 1, 2, 4, and 8 h. Each FA treatments used five incubation bottles as replications along with three blanks for each hour. Thus, total 90 incubation bottles were used in the present study.

### Sampling

In an assigned hour (0, 1, 2, 4, and 8 h, respectively), all bottles were withdrawn from the incubator, the gas pressure was quickly measured by a pressure transducer (Fisher Scientific, Traceable^TM^, Friendswood, TX, United States), and then, the bottles were placed into ice to stop microbial activity (Honkanen et al., 2012). All incubated rumen samples including blank were subsampled 1 mL for microbial quantification using PCR. The remain incubated rumen samples were transferred to 50 mL conical tube to separate sample residue and supernatant of incubated rumen buffer through centrifugation at 2,568 × *g* for 15 min (Supra 21k, Hanil Electric Corporation, Seoul, South Korea, with rotor A50S-6C No.6). The supernatant of incubated rumen buffer from centrifugation was subsampled 10 mL and stored for further analyses of rumen fermentation indices. For FA analysis, 2 mL rumen buffer was frozen at −70°C for 2 days and freeze-dried using Cascade Console Freeze Dry System (LABCONCO, FreeZone Plus 12 Liter, MO, United States) as the procedure to sample preparation according to previous studies (Paradhipta et al., 2020).

### Analyses

#### Greenhouse Gas Emissions and Fermentation Indices

The total gas volume was calculated from the gas pressure (psi) according to Mauricio et al. (1999). Gas samples were collected in vacutainer tubes without additives for CH_4_ and CO_2_ analysis using a multigas analyzer (Yes Plus LGA, Critical Environment Technologies, Canada Inc., Delta, BC, Canada). The results of CH_4_ and CO_2_ were expressed as milligrams per gram of fresh weight. For rumen fermentation indices, the subsampled supernatant measured the pH using an electric pH meter (SevenEasy, Mettler Toledo, Greifensee, Switzerland). After then, the supernatant was centrifuged at 21,500 × *g* for 15 min for the analyses of ammonia-N (NH_3_-N) and VFA. The NH_3_-N concentration was measured by distillation of the sample in a Buchi apparatus (B-342, BÜCHI, Flawil, Switzerland) followed by titration with 0.1 *N* H_2_SO_4_ in a burette according to Chaney and Marbach (1962). The concentrations of VFAs were determined using an HPLC system (L-2200, Hitachi, Tokyo, Japan) fitted with a UV detector (L-2400; Hitachi) and a column (Metacarb 87H; Varian, Palo Alto, CA, United States) as described by Muck and Dickerson (1988).

#### Fatty Acid Profiles

Two-step methylation procedure was used for the preparation of FA methyl esters (FAMEs), which was described by Jenkins et al. (2001). One milligram of internal standard (C19:0) was added to the previously freeze-dried sample to calculate the total FA concentration. The FAs were esterified by adding 2 mL of sodium methoxide (Sigma Aldrich, St. Louis, MO, United States), followed by vortexing and then incubating in a 50°C water bath for 10 min. After cooling for 5 min, 3 mL of 5% methanolic HCl was added followed by vortexing. The tubes were then incubated at 80°C for 10 min in a water bath. After incubation, the solution was allowed to cool for 7 min, and 1 mL of hexane and 7.5 mL of K_2_CO_3_ were consecutively added. The tubes were shaken and centrifuged at 9,861 × *g* at 4°C. The upper layer containing FAMEs was transferred to a 2-mL top crimp vial (Agilent Technology, Cranberry Township, PA, United States) with Pasteur pipettes (Hilgenberg, Strauchgraben, Malsfeld, Germany). The FAMEs were analyzed using a gas chromatograph (450-GC, Varian, Palo Alto, CA, United States) equipped with an autosampler (CP-8400, Varian, Palo Alto, CA, United States), a flame ionization detector, and a Varian capillary column (CP-Sil 88, Palo Alto, CA, United States, 100 m × 0.25 mm × 0.2 μm). Hydrogen was the carrier gas. The injector and detector were maintained at 230°C. The oven temperature was initially set at 120°C for 1 min, increased by 5°C/min up to 190°C, held at 190°C for 30 min, increased again by 2°C/min up to 220°C, and held at 220°C for 40 min. The peak of the samples was identified, and concentrations were calculated based on the retention time and peak area of known standards.

#### DNA Extraction and Real-Time PCR

The DNA was extracted from incubated rumen sample by physical disruption using a mini bead-beater (BioSpec Products, Bartlesville, OK, United States) followed by isolation and purification using a commercial DNA extraction kit (QIAamp DNA mini kit, Qiagen, Germantown, MD, United States). A 700-μL aliquot of homogenously incubated rumen sample was transferred into a 1.5-mL microcentrifuge tube, and 180 μL of buffer animal tissue lysis (ATL) and 20 μL of proteinase K (supplied in the QIAamp DNA Mini Kit) were added to the tube followed by vortexing. The tubes were then incubated at 56°C for 12 h for cell lysis in a heating block (HB-48, Daihan Scientific Co., Ltd., Seoul, South Korea). Next, the DNA purification protocol was followed as described in the manufacturer’s instruction manual (Qiagen, Germantown, MD, United States). The DNA concentrations were measured by using a NanoDrop Spectrophotometer (ND-1000, United States). The primer information of general bacteria, *F. succinogenes*, *R. flavefaciens*, *R. albus*, rumen methanogenic archaea, and ciliates (*Entodinium*) are given in Table 1. Species-specific real-time qPCR was performed using a Bio-Rad C1000 Touch^TM^ Thermal Cycler Real-Time PCR Detection System (CFX96^TM^ Real-Time System, Bio-Rad Laboratories, Inc., Hercules, CA, United States), with fluorescence detection by SYBR Green Real-time Master Mix (TOYOBO Co., Ltd., Osaka, Japan). The values of the cycle threshold (Ct) after real-time PCR were used to determine the fold change of different microbial populations relative to the respective blanks without FA treatments. The abundance of these microbes was expressed by the following equation: relative quantification = 2^–Δ*CT*(*Target*)–Δ*CT*(*Blank*)^, where Ct represents the threshold cycle. Rumen general bacteria were used as reference genes for internal controls according to Kim et al. (2012). All quantitative PCR mixtures (final volume of 20 μL) contained forward and reverse primers, SYBR Green Master Mix, DNA template, and sterilized distilled water. A negative control without template DNA was used in every qPCR assay for each primer.

**TABLE 1:** Oligonucleotide primers used for real-time PCR assay.

	**Sequence (5′-3′)**	**Size (bp)**	**References**
General bacteria	F-CGGCAACGAGCGCAACCC/R-CCATTGTAGCACGTGTGTAGCC	130	Denman and McSweeney (2006)
*Fibrobacter succinogenes*	F-GTTCGGAATTACTGGGCGTAAA/R-CGCCTGCCCCTGAACTATC	121	Denman and McSweeney (2006)
*Ruminococcus flavefaciens*	F-GAACGGAGATAATTTGAGTTTACTTAGG/R-CGGTCTCTGTATGTTATGAGGTATTACC	132	Denman and McSweeney (2006)
*Ruminococcus albus*	F-CCCTAAAAGCAGTCTTAGTTCG/R-CCTCCTTGCGGTTAGAACA	175	Koike and Kobayashi (2001)
Methanogenic archaea	F-TTCGGTGGATCDCARAGRGC/R-GBARGTCGWAWCCGTAGAATCC	140	Denman et al. (2007)
Ciliates (*Entodinium*)	F-GAG CTA ATA CAT GCT AAG GC/R-CCC TCA CTA CAA TCG AGA TTT AAG G	180	Skillman et al. (2006)

### Statistical Analyses

The experiment was a completely randomized design. All data were analyzed using the general linear model procedure of Statistical Analysis System (SAS) ver. 9.3 (SAS, 2000). Its model was *Y*_*ij*_ = μ + *T*_*i*_ + *e*_*ij*_, where *Y*_*ij*_ = response variable, μ = overall mean, *T* = effect of treatment *i*, and *e*_*ij*_ = error effect. In addition, rumen pH, NH_3_-N, acetate, propionate, CH_4_, CO_2_, rumen microbes, and major 18-carbon FAs were analyzed using the PROC MIXED procedure of SAS to test the significant levels of supplementary treatment, incubation hour, and the interaction between supplementary treatment and incubation hour. Its model was *Y*_*ijk*_ = μ + α*_*i*_* + β*_*j*_* + (αβ)*_*ij*_* + *e*_*ijk*_, where *Y*_*ijk*_ = response variable, μ = overall mean, α*_*i*_* = the effect of supplementary treatment, β*_*j*_* = the effect of incubation hour, (αβ)*_*ij*_* = the interaction effect between supplementary treatment and incubation hour, and *e*_*ijk*_ = error effect. Mean differences were tested for significance using Tukey’s test at *P* ≤ 0.05.

## Results

### Fatty Acid Profiles Before Incubation

The total FAs before incubation from LA, LNA, and Combo were 4.11, 3.87, and 3.77 mg/mL, respectively (Table 2). The concentrations of C18:2n-6 was highest (*P* < 0.001; 18.4 vs. 11.02 vs. 3.07%) in LA, followed by Combo and LNA, while the concentrations of C18:3n-3 was highest (*P* < 0.001; 6.78 vs. 3.72 vs. 0.26%) in LNA, followed by Combo and LA. The concentrations of C14:0, C14:1, C16:0, C16:1, C18:0, SFA, monounsaturated fatty acid (MUFA), and PUFA were higher (*P* < 0.05) in LNA than LA. The ratio of SFA to PUFA was higher (*P* = 0.005; 7.65 vs. 3.74 and 4.92) in LNA than LA and Combo. In addition, the ratio of n-6 to n-3 was higher (*P* < 0.001; 35.76 vs. 0.48 and 2.84) in LA than LNA and Combo.

**TABLE 2:** Effects of fatty acid supplementation on the fatty acid profiles of rumen buffer just before incubation.

	**Treatments**			**SEM**	***P* value**
	**LA**	**LNA**	**Combo**		
Total FAs (mg/mL)	4.11	3.87	3.77	0.293	0.356
C14:0 (% total FA)	4.14^b^	4.66^a^	4.41^ab^	0.176	0.009
C14:1 (% total FA)	1.11^b^	1.27^a^	1.21^ab^	0.056	0.014
C16:0 (% total FA)	21.65^b^	24.37^a^	22.94^ab^	0.789	0.004
C16:1 (% total FA)	0.60^b^	0.70^a^	0.65^ab^	0.034	0.010
C18:0 (% total FA)	45.58^b^	50.10^a^	47.31^ab^	2.050	0.035
C18:1*cis*-9 (% total FA)	6.63	7.34	7.11	0.399	0.099
C18:2n-6 (% total FA)	18.41^a^	3.07^c^	11.02^b^	0.893	<0.001
C18:3n-3 (% total FA)	0.26^c^	6.78^a^	3.72^b^	0.128	<0.001
C20:0 (% total FA)	0.65	0.70	0.68	0.035	0.178
C20:3n-6 (% total FA)	0.30	0.31	0.30	0.017	0.399
C22:6n-3 (% total FA)	0.26	0.28	0.27	0.015	0.232
C24:1 (% total FA)	0.41	0.42	0.37	0.041	0.186
SFAs (% total FA)	72.02^b^	79.83^a^	75.34^ab^	2.826	0.012
MUFAs (% total FA)	8.75^b^	9.73^a^	9.35^ab^	0.446	0.044
PUFAs (% total FA)	19.23^a^	10.44^b^	15.31^ab^	1.077	0.010
SFAs:PUFAs	3.74^b^	7.65^a^	4.92^b^	0.130	0.005
n-6:n-3	35.76^a^	0.48^b^	2.84^b^	0.709	<0.001

*^*a–c*^Means with different superscripts in the same row differ significantly (*P* < 0.05).LA, LNA, and Combo denote treatments containing pure C18:2n-6 FA, C183n-3 FA, and the equal mixture of these two, respectively. FAs, fatty acids; SFAs, saturated fatty acids; MUFAs, monounsaturated fatty acids; PUFAs, polyunsaturated fatty acids.*

### Fermentation Indices and Greenhouse Gas Emissions

After 8 h of incubation, the pH was lower (*P* = 0.034; 6.52 vs. 6.65) in LA than in Combo, while the pH in LNA was not different compared with that in the other treatments (Table 3). The NH_3_-N concentration was higher (*P* = 0.002; 33.46 vs. 30.38 and 30.38 mg/100 mL) in LA than in LNA and Combo after 8 h of incubation. The total VFA concentration was higher (*P* = 0.039; 85.22 vs. 78.93 mmol/L) in LA than in Combo, while the concentration in LNA did not differ compared with that in the other treatments. Among the individual VFAs, the concentrations of acetate, propionate, butyrate, isovalerate, and valerate remained unaffected. Only the concentration of isobutyrate was found to be higher (*P* < 0.001; 0.93 vs. 0.83 and 0.74%) in LA than in LNA and Combo. The total gas volume in LA was higher (*P* < 0.001; 35.43 vs. 31.13 and 30.29 mL/g) than that in LNA and Combo. The CH_4_ emissions were highest (*P* = 0.001; 55.50 vs. 46.60 vs. 40.20 mg/g) in LA, followed by LNA and then Combo. The CO_2_ gas concentration was higher in Combo than in LA and LNA (*P* = 0.042; 2.05 vs. 1.61 vs. 1.80 mg/g).

**TABLE 3:** Effects of fatty acid supplementation on the fermentation indices and greenhouse gas emissions of rumen buffer after 8 h of incubation.

	**Treatments**			**SEM**	***P* value**
	**LA**	**LNA**	**Combo**		
pH	6.52^b^	6.62^ab^	6.65^a^	0.070	0.034
NH_3_-N (mg/100 mL)	33.46^a^	30.38^b^	30.38^b^	1.199	0.002
Total VFAs (mmol/L)	85.22^a^	80.55^ab^	78.93^b^	3.916	0.039
Acetate (% molar)	47.02	46.56	46.86	0.466	0.316
Propionate (% molar)	23.57	23.78	23.53	0.612	0.791
Isobutyrate (% molar)	0.93^a^	0.83^b^	0.74^b^	0.055	<0.001
Butyrate (% molar)	14.50	14.11	14.30	0.356	0.254
Isovalerate (% molar)	6.66	6.43	6.43	0.212	0.174
Valerate (% molar)	4.31	4.88	4.64	0.688	0.443
Acetate:propionate	2.00	1.96	1.99	0.063	0.521
Total gas volume (mL/g DM)	35.43^a^	31.13^b^	30.29^b^	0.557	<0.001
CH_4_ (mg/g)	55.50^a^	46.60^b^	40.20^c^	2.420	0.001
CO_2_ (mg/g)	1.61^b^	1.80^b^	2.05^a^	0.162	0.042

*^*a–c*^Means with different superscripts in the same row differ significantly (*P* < 0.05).LA, LNA, and Combo denote treatments containing pure C18:2n-6 FA, C183n-3 FA, and the equal mixture of these two, respectively.*

**FIGURE 1:**
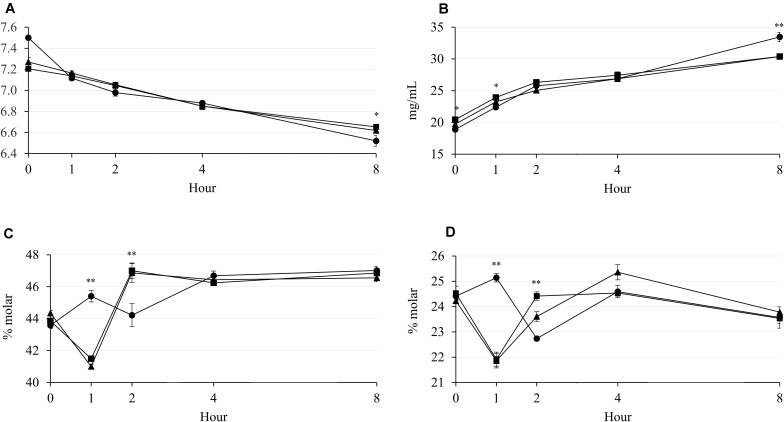
Changes in rumen pH **(A)** and concentrations of NH_3_-N **(B)**, acetate **(C)**, and propionate **(D)** during rumen incubation with FAs for 8 h. Diet with C18:2n-6, LA (filled circles); diet with C18:3n-3, LNA (filled triangles); and diet with the mixture of C18:2n-6 and C18:3n-3 at a ratio of 1:1 (filled squares). The respective significance levels of the supplementary treatment, incubation period, supplementary treatment × incubation period, and SEM for pH, NH_3_-N, acetate, and propionate are *P* < 0.001, *P* = 0.054, *P* = 0.041, and SEM = 0.057; *P* < 0.001, *P* = 0.115, *P* < 0.001, and SEM = 1.064; *P* < 0.001, *P* = 0.071, *P* < 0.001, and SEM = 0.715; and *P* < 0.001, *P* = 0.037, *P* ≤ 0.001, and SEM = 0.487. Different symbols at the same hour differ significantly. ^∗^*P* < 0.05; ^∗∗^*P* < 0.01.

**FIGURE 2:**
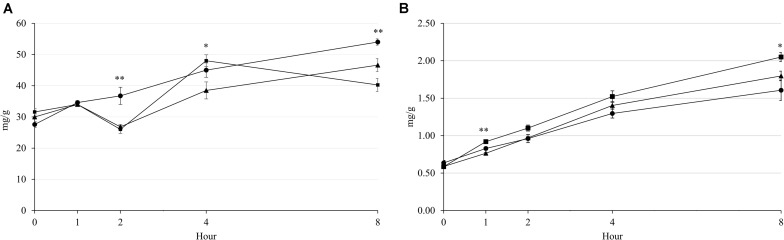
Changes in CH_4_ **(A)** and CO_2_ **(B)** emissions during rumen incubation with FAs for 8 h. Diet with C18:2n-6, LA (filled circles); diet with C18:3n-3, LNA (filled triangles); and diet with the mixture of C18:2n-6 and C18:3n-3 at a ratio of 1:1 (filled squares). The respective significance levels of the supplementary treatment, incubation period, supplementary treatment × incubation period, and SEM for CH_4_ and CO_2_ are *P* < 0.001, *P* = 0.001, *P* < 0.001, and SEM = 3.021 and *P* < 0.001, *P* < 0.001, *P* = 0.005, and SEM = 91.69. Different symbols at the same hour differ significantly. ^∗^*P* < 0.05; ^∗∗^*P* < 0.01.

The rumen pH was decreased by increasing incubation hour, but supplementary treatment had no effect from 0 to 4 h of incubation (Figure 1A). The NH_3_-N concentration was increased by incubation hour in all supplementary treatments, which Combo presented higher concentration than LA at 0 h (*P* = 0.043; 20.44 vs. 18.90 mg/100 mL) and 1 h (*P* = 0.032; 23.94 vs. 22.40 mg/100 mL) of incubation (Figure 1B). The acetate and propionate concentrations were affected by the supplementary treatment at 1 and 2 h of incubation, but the patterns of concentration change over hour were cubical pattern (Figures 1C,D). Furthermore, LA resulted in highest concentrations of acetate (*P* = 0.001; 45.41 vs. 41.01 and 41.49%) and propionate (*P* < 0.001; 25.14 vs. 21.86 and 21.92%) at 1 h, then had lowest (*P* < 0.01) concentrations of acetate (*P* < 0.001; 44.22 vs. 46.86 and 47.00%) and propionate (*P* = 0.001; 22.74 vs. 23.61 and 24.42%) at 2 h of incubation. There were interaction effects between the supplementary treatment and hour on rumen pH (*P* = 0.041) and NH_3_-N (*P* < 0.001), acetate (*P* < 0.001), and propionate (*P* < 0.001) concentrations, which might be a reason for the cubical patterns of these results during incubation. The concentration of CH_4_ was increased by increasing incubation hour in all supplementary treatments (Figure 2A). LA had highest (*P* < 0.01; 3.68 vs. 2.68 and 2.60 mg/g) CH_4_ concentration at 2 h of incubation, while LA and Combo presented higher (*P* < 0.05; 4.50 and 4.80 vs. 3.85 mg/g) concentration than LNA at 4 h of incubation. Similar to CH_4_, concentration of CO_2_ was also increased by increasing incubation hour, which Combo and LA presented higher (*P* < 0.05; 0.83 and 0.92 vs. 0.76 mg/g) concentration than LNA at 1 h or incubation (Figure 2B). Additionally, there were interaction effects between the supplementary treatment and hour on the CH_4_ (*P* < 0.001) and CO_2_ (*P* = 0.005) concentrations.

### Microbial Populations

It was observed that methanogenic archaea were less abundant (*P* = 0.03; 0.74 vs. 0.96 and 0.96) in LNA than in LA and Combo (Table 4). Rumen ciliates were less abundant (*P* = 0.03; 0.20 vs. 0.41 of fold change) in LA than in LNA, respectively, while the population in Combo had no difference compared with that in the other treatments. Among fibrolytic bacteria, *R. albus* was observed to be more abundant (*P* = 0.005; 0.17 vs. 0.09 and 0.10 of fold change) in LA than in LNA and Combo. *F. succinogenes* and *R. flavefaciens* remained unaffected by the treatments at 8 h of incubation.

**TABLE 4:** Effects of fatty acid supplementation on microbial populations of rumen buffer after 8 h of incubation (fold change unit).

	**Treatments**			**SEM**	***P* value**
	**LA**	**LNA**	**Combo**		
Methanogenic archaea	0.96^a^	0.74^b^	0.96^a^	0.093	0.039
Rumen ciliates	0.20^b^	0.41^a^	0.22^ab^	0.079	0.036
**Fibrolytic bacteria**					
*F. succinogenes*	0.67	0.52	0.56	0.064	0.063
*R. albus*	0.17^a^	0.09^b^	0.10^b^	0.021	0.005
*R. flavefaciens*	0.50	0.41	0.36	0.105	0.326

*^*a–b*^Means with different superscripts in the same row differ significantly (*P* < 0.05).LA, LNA, and Combo denote treatments containing pure C18:2n-6 FA, C183n-3 FA, and the equal mixture of these two, respectively.*

**FIGURE 3:**
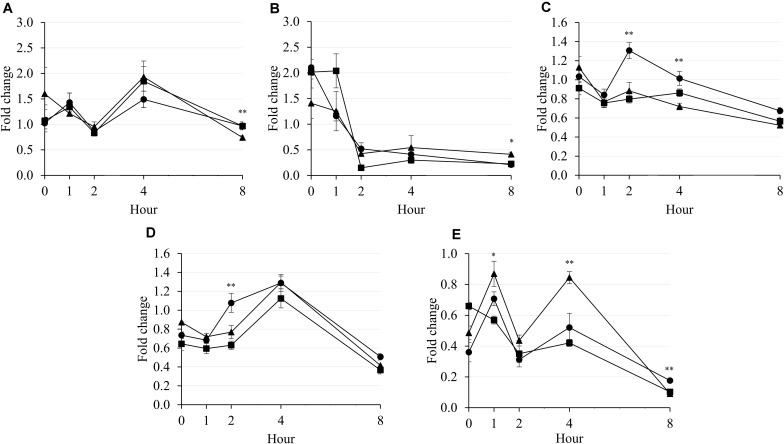
Fold change in methanogenic archaea **(A)**, rumen ciliate **(B)**, *F. succinogenes* **(C)**, *R. flavefaciens* **(D)**, and *R. albus* **(E)** compared with that of the blank during rumen incubation with FAs for 8 h. Diet with C18:2n-6, LA (filled circles); diet with C18:3n-3, LNA (filled triangles); and diet with the mixture of C18:2n-6 and C18:3n-3 at a ratio of 1:1 (filled squares). The respective significance levels of the supplementary treatment, incubation period, supplementary treatment × incubation period, and SEM for methanogenic archaea, rumen ciliates, *F. succinogenes*, *R. flavefaciens*, and *R. albus* are *P* = 0.255, *P* = 0.157, *P* = 0.492, and SEM = 2.046; *P* < 0.001, *P* = 0.458, *P* = 0.019, and SEM = 0.344; *P* < 0.001, *P* < 0.001, *P* = 0.003, and SEM = 0.124; *P* < 0.001, *P* = 0.032, *P* = 0.301, and SEM = 0.168; and *P* < 0.001; *P* = 0.001, *P* < 0.001, and SEM = 0.099. Different symbols at the same hour differ significantly. ^∗^*P* < 0.05; ^∗∗^*P* < 0.01.

It was observed that the population of methanogenic archaea was not affected by supplementary treatment during 4 h of incubation (Figure 3A). The population of rumen ciliates dropped drastically from 0 to 2 h of incubation in all supplementary treatments (Figure 3B). The population of *F. succinogenes* seemed to decrease by increasing incubation hour (Figure 3C). Furthermore, LA presented higher *F. succinogenes* population than LNA and Combo at 2 h (*P* = 0.003; 1.31 vs. 0.88 and 0.80) and 4 h (*P* = 0.001; 1.01 vs. 0.72 and 0.86) of incubation. In general, *R. flavefaciens* population increased during 4 h of incubation in all supplementary treatments, then decreased after that (Figure 3D). The *R. flavefaciens* population was higher (*P* = 0.007; 1.08 vs. 0.77 and 0.63) in LA and presented higher population than in LNA and Combo at 2 h of incubation. The population of *R. albus* was higher in LNA than in LA and Combo at 1 h (*P* = 0.015; 0.87 vs. 0.71 and 0.57) and 4 h (*P* = 0.008; 0.84 vs. 0.52 and 042) of incubation (Figure 3E). Generally, all supplementary treatments seemed to decrease the population of *R. albus* after 8 h of incubation. An interaction between the supplementary treatment and hour was reported for rumen ciliates (*P* = 0.019), *F. succinogenes* (*P* = 0.003), and *R. albus* (*P* < 0.001), which caused cubical patterns of those microbes during incubation.

### Fatty Acid Profiles After Incubation

The concentrations of total FAs and MUFAs were found to be unaffected (*P* > 0.05) by the treatments after 8 h of incubation (Table 5). The concentrations of C14:1 (*P* = 0.001; 1.45 and 1.44 vs. 1.30%) and C16:1 (*P* = 0.001; 0.89 and 0.87 vs. 0.77%) were higher in LNA and Combo than in LA. Otherwise, the concentrations of C18:0 (*P* = 0.008; 56.62 vs. 55.45 and 55.28%) and C20:3n-6 (*P* = 0.001; 0.36 vs. 0.33 and 0.33%) were higher in LA than in LNA and Combo. On the other hand, the C18:2n-6 concentration was higher (*P* < 0.001; 3.05 and 3.66 vs. 1.82%) in LA and Combo than in LNA. The C18:3n-3 concentration was higher (*P* < 0.001; 1.88 vs. 1.26 vs. 0.20%) in LNA, followed by Combo and then LA. LA and LNA produced higher concentrations of C20:0 (*P* = 0.001; 0.80 and 0.78 vs. 0.76%) and C24:1 (*P* = 0.005; 0.46 and 0.46 vs. 0.42%) than did Combo. LA had a higher SFA concentration (*P* = 0.005; 86.87 vs. 85.11%) than that in Combo, while the concentration of SFA in LNA was not different compared with that in the other treatments. Combo had the highest PUFA concentration (*P* = 0.002; 5.58 vs. 3.96 and 4.37%), but it had the lowest ratio of SFAs to PUFAs (*P* = 0.002; 15.25 vs. 21.93 and 19.69) compared with those of the other treatments.

**TABLE 5:** Effects of fatty acid supplementation on the fatty acid profiles of rumen buffer after 8 h of incubation.

	**Treatments**			**SEM**	***P* value**
	**LA**	**LNA**	**Combo**		
Total FAs (mg/mL)	4.03	3.21	3.45	0.855	0.334
C14:0 (% total FA)	4.60	4.79	4.70	0.119	0.078
C14:1 (% total FA)	1.30^b^	1.45^a^	1.44^a^	0.055	0.001
C16:0 (% total FA)	24.86	25.06	24.37	0.447	0.074
C16:1 (% total FA)	0.77^b^	0.89^a^	0.87^a^	0.040	0.001
C18:0 (% total FA)	56.62^a^	55.45^b^	55.28^b^	0.502	0.008
C18:1*cis*-9 (% total FA)	6.64	6.74	6.58	0.294	0.672
C18:2n-6 (% total FA)	3.05^a^	1.82^b^	3.66^a^	0.385	<0.001
C18:3n-3 (% total FA)	0.20^c^	1.88^a^	1.26^b^	0.294	<0.001
C20:0 (% total FA)	0.80^a^	0.78^a^	0.76^b^	0.013	0.001
C20:3n-6 (% total FA)	0.36^a^	0.33^b^	0.33^b^	0.009	0.001
C22:6n-3 (% total FA)	0.34	0.33	0.33	0.013	0.205
C24:1 (% total FA)	0.46^a^	0.46^a^	0.42^b^	0.019	0.004
SFAs (% total FA)	86.87^a^	86.08^ab^	85.11^b^	0.684	0.005
MUFAs (% total FA)	9.17	9.54	9.31	0.283	0.156
PUFAs (% total FA)	3.96^b^	4.37^b^	5.58^a^	0.565	0.002
SFAs:PUFAs	21.93^a^	19.69^a^	15.25^b^	1.295	0.002
n-6:n-3	6.27^a^	1.00^b^	2.57^c^	0.376	<0.001

*^*a–c*^Means with different superscripts in the same row differ significantly (*P* < 0.05).LA, LNA, and Combo denote treatments containing pure C18:2n-6 FA, C18:3n-3 FA, and the equal mixture of these two, respectively. FAs, fatty acids; SFAs, saturated fatty acids; MUFAs, monounsaturated fatty acids; PUFAs, polyunsaturated fatty acids.*

**FIGURE 4:**
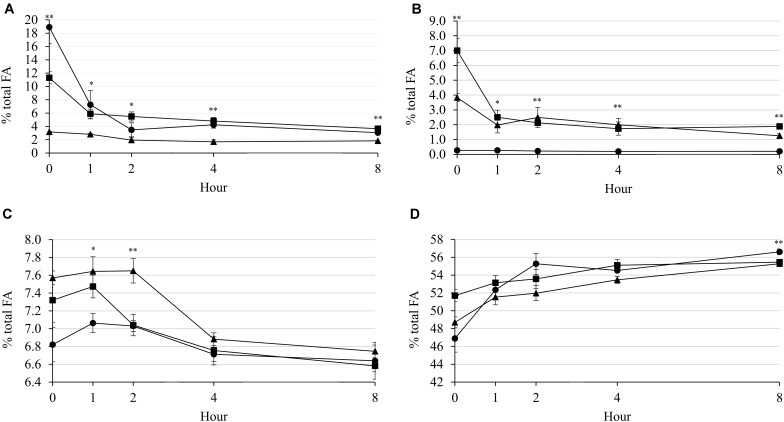
Changes in C18:2n-6 **(A)**, C18:3n-3 **(B)**, C18:1*cis*-9 **(C)**, and C18:0 **(D)** concentrations (%, FA) during rumen incubation with FAs for 8 h. Diet with C18:2n-6, LA (filled circles); diet with C18:3n-3, LNA (filled triangles); and diet with the mixture of C18:2n-6 and C18:3n-3 at a ratio of 1:1 (filled squares). The respective significance levels of the supplementary treatment, incubation period, supplementary treatment × incubation period, and SEM for concentrations of C18:2n-6, C18:3n-3, C18:1n-9, and C18:0 are *P* < 0.001, *P* < 0.001, *P* < 0.001, and SEM = 1.915; *P* < 0.001, *P* < 0.001, *P* < 0.001, and SEM = 1.111; *P* < 0.001, *P* = 0.001, *P* = 0.218, and SEM = 0.306; and *P* < 0.001, *P* < 0.001, *P* = 0.036, and SEM = 1.741. Different symbols at the same hour differ significantly. ^∗^*P* < 0.05; ^∗∗^*P* < 0.01.

Generally, applications of LA and Combo decreased concentration of C18:2n-6 during incubation, while applications of LNA and Combo decreased concentration of C18:3n-3. At 1 h (*P* = 0.012; 7.61 vs. 5.61 vs. 2.68%), 2 h (*P* = 0.019; 3.33 and 3.48 vs. 1.96%), and 4 h (*P* = 0.001; 4.24 and 4.81 vs. 1.69%) were observed that the C18:2n-6 concentration was higher in LA and Combo than in LNA (Figure 4A). However, the C18:3n-3 concentration was higher in LNA and Combo than in LA at 1 h (*P* = 0.015; 2.51 and 1.98 vs. 0.27%), 2 h (*P* = 0.012; 2.13 and 2.49 vs. 0.23%), and 4 h (*P* = 0.008; 1.74 and 2.00 vs. 0.20%) (Figure 4B). All supplementary treatments seemed to increase C18:1*cis*-9 concentration during 2 h of incubation, and then decreased it after that (Figure 4C). The concentration of C18:1*cis*-9 was higher (*P* = 0.028; 7.64 and 747 vs. 7.06) in LNA and combo at 1 h. At 2 h, LNA had the highest (*P* = 0.008; 7.65 vs. 7.03 and 7.04%) concentration of C18:1*cis*-9. The C18:0 concentration was not affected by the supplementary treatment during incubation (Figure 4D). The concentration of C18:0 was increased by incubation hour in all supplementary treatments. Interaction effects between the supplementary treatment and hour were observed on C18:2n-6 (*P* < 0.001), C18:3n-3 (*P* < 0.001), and C18:0 (*P* = 0.036).

## Discussion

### Rumen Fermentation, Greenhouse Gases, and Microbes

The increases of NH_3_-N and total VFA concentrations and total gas volume with a decrease of rumen pH in LA indicated a higher relative rumen fermentation rate by LA compared with LNA or Combo. The reason for the high VFA concentration and total gas volume in LA could be supported by the higher total amount of fibrolytic bacteria in LA than in LNA and Combo (Table 4), which increased rumen digestion. It was found in Martin et al. (2006) that supplementary treatment with C18:3n-3 at either 100 or 50% could depress degradations of organic matter and fiber in the rumen. This previous study also reported that the population of total fibrinolytic bacteria was lower in LNA and Combo than in LA. In addition, LA had the highest acetate and propionate concentrations at 1 h of incubation, and then presented the lowest at 2 h of incubation. The reason of these results was unknown in the present study. However, on the other side, LA increased the CH_4_ emissions. In agreement with the present study, Zhang et al. (2008) observed higher total gas production and CH_4_ concentration by supplementary C18:2n-6 compared with C18:3n-3. Combo had the lowest CH_4_ emissions in the present study, even though it presented a high population of methanogenic archaea. In addition, supplementary n-9 FA such as oleic acid was reported to reduce methane production in an *in vitro* study (Wu et al., 2016). The reason for the low CH_4_

emissions by Combo was not clear. However, this observation supported the result of the highest CO_2_ concentration occurring in Combo followed by LNA and LA numerically, which could indicate a low conversion rate of CO_2_ to CH_4_ by methanogenic archaea (Wolin et al., 2007; Tapio et al., 2017). Combination of n-3 and n-6 FAs might effectively inhibit methanogenic archaea to utilize free CO_2_ during methanogenesis process, which could reduce methane gas emission without decrease its population. However, the further investigations need to be conducted.

Supplementary PUFAs are considered to depress methanogenesis (Fievez et al., 2003). Microbial DNA analysis showed that methanogenic archaea were suppressed only by LNA, while ciliates were suppressed by both LA and Combo. Hristov et al. (2013) reported that both C18:2n-6 and C18:3n-3 were effective to decrease rumen ciliate. Supporting the result of the present study, other previous study reported that C18:2n-6 was more effective than C18:3n-3 to decrease ciliates. However, effectiveness of C18:2n-6 against C18:3n-3 to inhibit ciliates could be influenced by supplementation levels on rumen fluid (Zhang et al., 2008). Combo also contained C18:2n-6 that could present a similar result of ciliates with LA in the present study. In the present study, the observed lower population of methanogenic archaea in LNA than in other treatments (Table 3) is in agreement with other findings (Fievez et al., 2003; Zhang et al., 2008). Methanogenic archaea and rumen ciliate might had different sensibility with each pure essential FA that caused the decrease of methanogenic archaea in LNA and rumen ciliate in LA and Combo. Along with methanogenic archaea, rumen ciliates are responsible for CH_4_ emissions in the rumen (Whitelaw et al., 1984). Rumen ciliates are associated with ecto- and endosymbiotic methanogenic archaea (Finlay et al., 1994). Therefore, the degradation of fiber and suppressive effects of FAs on methanogens and ciliates might have occurred and contributed to the CH_4_ emissions in the present study. Increased fiber degradation in the rumen is also responsible for an increase of enteric CH_4_ emission (Hristov et al., 2013). Decreased methanogenesis sometimes shifts rumen fermentation from acetogenic to propionigenic (Wettstein et al., 2000) because of increased hydrogen availability for propionate synthesis. However, this relation was only observed at 4 h of incubation, when CH_4_ production was lowest but propionate production was numerically highest in the LNA treatment (Figures 1D, 2A).

As shown in Figure 3, the population of methanogenic archaea in LNA tended to decrease over 8 h of incubation, while the other treatments showed similar populations before and after incubation. LNA has a higher degree of unsaturation than LA. Although most long-chain PUFAs could exert toxic effects on rumen microbes (Maia et al., 2007), greater effects were reported with a higher degree of unsaturation as well as a higher applied dose (Zhang et al., 2008). Both the direct toxic effects of the FA and decreased amount of ruminal hydrogen in LNA could negatively affect methanogens, compared with the effects in the other treatments. A higher degree of unsaturation in LNA consumed more hydrogen for biohydrogenation of the FA (Zhang et al., 2008). However, it was reported that only 1 to 2% of the metabolic hydrogen in the rumen is used for the purpose of biohydrogenation (Jenkins et al., 2008). Therefore, the CH_4_ emissions by LNA were reported to be lower than those by LA in the present study. On the other hand, LNA has previously been shown to be toxic to cellulolytic bacteria, particularly *F. succinogenes*, *R. albus*, and *R. flavefaciens* (Maia et al., 2007). This previous study supported the results of the present study, which reported lower populations of *R. albus* and *F. succinogenes* (numerically, *P* = 0.063) bacteria in LNA than in LA after 8 h of incubation. Martin et al. (2006) explained that the negative effects of PUFAs on fibrolytic bacteria were not only the result of direct toxicity of PUFAs themselves but also indirectly related to the inhibition of methanogens. Nevertheless, compared with LA, LNA presented higher *R. albus* populations at 1 and 4 h of incubation in the present study, but the reason for this result was not clear. The inhibition of methanogens leads to the accumulation of free hydrogen in the rumen, which can negatively affect the growth of cellulolytic bacteria (Wolin et al., 2007). The LNA treatment inhibited methanogenic archaea to a greater extent and consequently exerted maximum negative effects on fibrolytic bacteria overall compared with the effects of the other treatments. Generally, populations of all microbes decreased after 8 h of incubation because substrate was completely degraded and indicated the end of ruminal fermentation. In addition, it also supported the results of major C18 FAs, which also decrease after 8 h of incubation (Figure 4).

### Fatty Acid Profiles in the Rumen

It is well known that unsaturated FAs undergo extensive biohydrogenation in the rumen by microbes (Beam et al., 2000; Petri et al., 2014). However, the rate of biohydrogenation might vary depending on the source or form of FA supplied (Carriquiry et al., 2008; Honkanen et al., 2012; Petri et al., 2014). In the present study, pure C18:2n-6 FA and C18:3n-3 FA were used either alone or in combination in equal ratios, and the results suggested less FA degradation in the combined treatment. At 0 h of incubation, the highest concentration of C18:2n-6 in LA and the highest concentration of C18:3n-3 in LNA were expected before considering the respective FAs were added accordingly in those treatments (Table 2). Furthermore, the application of mixed n-6 and n-3 FAs increased the concentrations of both C18:2n-6 FA and C18:3n-3 FA in the Combo treatment. Supplementary C18:2n-6 or C18:3n-3 did not have effect on total FA, but it could presented a minor change on proportion of FA profiles in the diet that could be a reason for results for C14:0, C14:1, C16:0, C16:1, and C18:0 at 0 h. Although the PUFAs were biohydrogenated after 8 h of incubation, the concentration patterns of remained C18:2n-6 FA and C18:3n-3 FA was similar (Table 5) to those in the beginning. This result indicated that the supplementation of these FAs could ensure their existence at an increased rate in the rumen, at least up to 8 h, which is in agreement with Carriquiry et al. (2008). Moreover, the use of these FAs in combination exerted inhibition of biohydrogenation to some extent, in contrast to the individual FA treatments. Lower C18:0 FA and total SFA concentrations and moderate C18:3n-3 concentrations and n-6 to n-3 ratios but higher C18:2n-6 FA and total PUFA concentrations in Combo indicated restricted biohydrogenation in this treatment (Table 5). This indication is more pronounced in Figure 4. This figure shows that from 0 to 2 h of incubation, approximately at 81.6% of linoleic acid was biohydrogenated in the LA treatment, and only 16.2% remained at the end of incubation (8 h). In Combo, 51.4% was biohydrogenated at 2 h, and 32.4% of C18:2n-6 remained at the end of incubation. In the case of C18:3n-3 FA, biohydrogenation was approximately at 64.2 vs. 48.3% in LNA vs. Combo, respectively at the first hour of incubation. And at the end, the remaining C18:3n-3 concentrations were approximately at 26.8 vs. 32.9%. In contrast, the concentration of C18:0 FA, the final end product of biohydrogenation of 18-carbon PUFAs, remained lower in Combo throughout the incubation. These results indicated the partial restriction of biohydrogenation of PUFAs when used in mixtures rather than individually.

## Conclusion

The results indicated that better fermentation occurred in the LA treatment than in the LNA and Combo treatments, as expressed by the higher total gas volume and NH_3_-N and total VFA concentrations. However, the CH_4_ emissions were suppressed in Combo, followed by LNA and LA. Population of rumen ciliates was decreased by LA, but populations of methanogenic archaea and *R. albus* were decreased by LNA compared with the other treatments. Adding C18:2n-6 and C18:3n-3 in the diet can increase their proportion in the ruminal contents. A lower ratio of n-6 to n-3 can be achieved by dietary supplementation with n-3 FAs. The CH_4_ emissions and the extent of biohydrogenation were reduced when these FAs were used in combination rather than individually, as indicated by the lower SFA but higher PUFA concentrations in the Combo treatment than in the other treatments. Therefore, the present study recommended Combo treatment for ruminant because it was more effective to reduce enteric methane emissions and resist biohydrogenation in the rumen compared with LA and LNA.

## Data Availability Statement

The raw data supporting the conclusions of this article will be made available by the authors, without undue reservation.

## Ethics Statement

The animal study was reviewed and approved by Animal Ethics Committee of Gyeongsang National University, South Korea (GNU-191011-E0050).

## Author Contributions

SMA, DHK, and SCK designed the study. SMA, DHK, DHVP, and SCK wrote and edited manuscript. All authors performed the experiment and analyzed the data.

## Conflict of Interest

The authors declare that the research was conducted in the absence of any commercial or financial relationships that could be construed as a potential conflict of interest.
